# Concentration of mitochondrial DNA mutations by cytoplasmic transfer from platelets to cultured mouse cells

**DOI:** 10.1371/journal.pone.0213283

**Published:** 2019-03-04

**Authors:** Kaori Ishikawa, Kohei Kobayashi, Akihito Yamada, Moe Umehara, Toshihiko Oka, Kazuto Nakada

**Affiliations:** 1 Faculty of Life and Environmental Sciences, University of Tsukuba, Tennodai, Tsukuba, Ibaraki, Japan; 2 Graduate School of Life and Environmental Sciences, University of Tsukuba, Tennodai, Tsukuba, Ibaraki, Japan; 3 Department of Life Science, Rikkyo University, Nishi-Ikebukuro, Toshima-ku, Tokyo, Japan; University of Parma, ITALY

## Abstract

Accumulation of mutations in mitochondrial DNA (mtDNA) is thought to be responsible for mitochondrial, and other, diseases and biological phenomena, such as diabetes, cancer, neurodegenerative diseases, and aging. Mouse models may elucidate the relationship between mutations in mtDNA and these abnormalities. However, because of the difficulty of mtDNA manipulation, generation of mouse models has not sufficiently progressed to enable such studies. To overcome this difficulty and to establish a source of diverse mtDNA mutations, we here generated cultured mouse cells containing mtDNA derived from an mtDNA mutator mouse that accumulates random mtDNA mutations with age. Mutation analysis of the obtained transmitochondrial cytoplasmic hybrid cells (cybrids) revealed that the cells harbored diverse mtDNA mutations occurring at a higher frequency than in mouse tissues, and exhibited severe respiration defects that would be lethal in tissues or organs. Abnormal respiratory complex formation and high stress on the mitochondrial protein quality control system appeared to be involved in these severe respiration defects. The mutation rates of the majority of highly accumulated mutations converged to either approximately 5%, 10%, or 40%, suggesting that these mutations are linked on the respective mtDNA molecules, and mtDNA in cybrid cells likely consisted of mtDNA molecules clonally expanded from the small population of introduced mtDNAs. Thus, the linked mutations in these cybrid cells cannot be evaluated individually. In addition, mtDNA mutations homologous to confirmed pathogenic mutations in human were rarely observed in our generated cybrids. However, the transmitochondrial cybrids constitute a useful tool for concentrating pathogenic mtDNA mutations and as a source of diverse mtDNA mutations to elucidate the relationship between mtDNA mutations and diseases.

## Introduction

Mitochondria produce the majority of ATP required by the body by oxidative phosphorylation (OXPHOS). Mitochondrial DNA (mtDNA), the unique mitochondrial genome, encodes 13 polypeptides—the subunits of respiratory complexes, and rRNA and tRNA molecules needed for translation of these polypeptides. Mutations in mtDNA result in reduced ATP production because they lead to abnormal structure of respiratory chain subunits or reduced translation of mitochondrial proteins, and underlie various disorders, termed mitochondrial diseases [1].

Until now, many mutations in mtDNA have been reported as candidate causative mutations of mitochondrial diseases [2]. All pathogenic mtDNA mutations result in reduced ATP production as a primary phenotype. However, the resultant disease phenotypes are diverse, and the mechanisms of expression of disease phenotypes are not well known. The most effective approach to elucidate these mechanisms is generation and analysis of animal models of disease, in which an animal harbors a mutation that corresponds to a disease of interest. However, because techniques for artificial manipulation and mutagenesis of mtDNA are not well established, generating animal models of disease corresponding to each human mitochondrial disease phenotype is very difficult.

Instead of using artificial mutagenesis, we have previously concentrated native mutations that exist in mouse cell lines at a very low frequency, and we then generated some mouse models of mitochondrial diseases [3–6]. However, this approach limits the array of mtDNA mutations as a source of mouse models of disease.

To overcome this difficulty, we focused on the mtDNA of “mutator mice”. Mutator mice have been generated by disrupting the proofread function of DNA polymerase γ (PolG), the only DNA polymerase for mtDNA [7, 8]. These mice accumulate random mtDNA mutations attributed to replication errors with age. Such diverse random mtDNA mutations possibly comprise mutations corresponding to certain human pathogenic mutations or novel pathogenic mutations. Hence, mtDNA of mutator mice is an attractive starting material for exploring pathogenic mtDNA mutations. However, the research group that created the mutator mice reported a strong elimination of non-synonymous changes in protein-coding genes and loss of variation of mutations in subsequent generations after crossing a mutator female with a wild-type male [9]. If the selective pressure that eliminates harmful mutations is operational in these mice, it would be difficult to concentrate certain pathogenic mtDNA mutations in mouse by repeated crossing.

To address this challenge, we conceived an idea of using a cultured cell line as a research tool. For example, although A3243G, the most frequent pathogenic mutation in mtDNA in human, is observed only as a heteroplasmy (mixture of mutant and normal mtDNA) in patients [10], it can exist as a homoplasmy (pure mutant mtDNA) in a cultured cell line [11]. This behavior is also common for other pathogenic mtDNA mutations. Furthermore, while mouse or human lacking mtDNA cannot exist, mtDNA-less cells (ρ^0^ cells) can be established and maintained in nutrient-rich medium supplemented with pyruvate and uridine [12, 13]. These observations suggest that cultured cell lines are resistant to severe respiration defects, and show higher tolerance to harmful pathogenic mtDNA than cells in tissue. If that indeed is the case, it should be possible to concentrate pathogenic mtDNA mutations using a cell line harboring mtDNA derived from mutator mouse.

In the current study, we established a transmitochondrial cytoplasmic hybrid (cybrid) cell line that harbors mtDNA containing random mutations by introducing platelet mtDNA from mutator mouse into mouse ρ^0^ cells. By evaluating respiratory function and the mtDNA sequence, we investigated whether mtDNA molecules containing random mutations might serve as a starting material for the generation of novel mouse models of disease. Further, we evaluated the difference in mtDNA mutation tolerance between mouse tissue and a cultured cell line, and the potential mechanisms that link respiration defects and random mtDNA mutations.

## Materials and methods

### Cells and cell culture

B82 is a fibrosarcoma cell line (ECACC 08062522) derived from the L929 fibroblast line established from C3H/An mouse. B82, ρ^0^B82 [13], and cybrids were grown at 37°C with 5% CO_2_ in RPMI 1640 medium (Nissui, Tokyo, Japan) containing 10% FBS (Sigma-Aldrich, St. Louis, MO), uridine (50 mg/mL), and pyruvate (0.1 mg/mL). All experiments were performed in accordance with the institutional guidelines.

### Mice

The control C57BL/6J mouse was purchased from a breeder (CLEA Japan, Tokyo, Japan). The mutator mouse line with C57BL/6J nuclear background was generated in our previous study [14]. All animal care and use were approved by the Institutional Animal Care and Use Committee of the University of Tsukuba.

### Generation of transmitochondrial cybrids

As mtDNA donors, 10-week-old male C57BL/6J mouse and 10-month-old male *PolG*^mut/mut^ mutator mouse were used. Blood samples (approximately 1 mL) were taken from each mouse under anesthesia and then centrifuged at 300 × *g* for 5 min. The collected platelet-rich supernatant was combined with 1 × 10^4^ ρ^0^B82 cells, and fused using the polyethylene glycol (PEG 1,500, Roche, Basel, Switzerland) method. Unfused nuclear donor ρ^0^B82 cells were eliminated by incubation in a selection medium lacking uridine and pyruvate. B82mtΔ cells have been generated previously [3]. Detailed procedures for the generation of transmitochondrial cybrids are described elsewhere [15]. The replacement of mtDNA in cybrids was confirmed by PCR-restriction fragment length polymorphism. Briefly, to distinguish the polymorphism at position 9,348 (G in C57BL/6J mouse and A in C3H/An mouse), mtDNA was purified using an mtDNA extractor CT kit (Wako, Osaka, Japan), and the 9,320–9,479-bp region was amplified by PCR. Forward primer *5′-AGC ATG ATA CTG ACA TTT TGT AGA CcT-3′* (lowercase letter indicates the mismatch site) and reverse primer *5′-GAT AAC AGT GTA CAG GTT AAT TAC TCT CTT CTG-3′* were used. Combination of the PCR-generated mismatch with the specific polymorphism created a restriction site for *Xsp* I in C57BL/6J mtDNA, such that *Xsp* I digestion generated 134-bp and 26-bp fragments, and an intact 160-bp amplicon in C3H/An mtDNA. Cybrid cells were cultured for at least 2 months after fusion before mtDNA sequence analysis.

### Biochemical determination of respiratory enzyme activity

Mitochondrial fraction was prepared from homogenized cell lysates, and mitochondrial respiratory complex activities were determined. To determine the activities of complexes I + III, II + III, and IV, NADH and oxidized cytochrome *c*, sodium succinate and oxidized cytochrome *c*, and reduced cytochrome *c* were added as substrates, respectively. Reduction (I + III, II + III) and oxidation (IV) of cytochrome *c* were detected as a change in sample absorbance at 550 nm. Mean reaction rates for B82mtB6 and B82mt*PolG*^mut/mut^ cells were calculated, and are reported as relative values, with B82mtB6 values considered to be 100%.

### Cytochrome *c* oxidase (COX)/succinate dehydrogenase (SDH) staining

Cytochemical analysis of COX and SDH activity was performed as described previously [16], using coverslips with growing cells.

### Deep-sequence analysis of mtDNA

mtDNA was extracted from cybrid cells by using an mtDNA extractor CT kit (Wako), electrophoresed on agarose gel, and purified by using the FastGene Gel/PCR Extraction Kit (Nippon Genetics, Tokyo, Japan). To compare the mtDNA populations between cybrids and parental platelets, platelets were prepared from the littermate of each mtDNA-donor mouse as described above at the same timing of the generation of cybrids, and mtDNA was extracted by using a Gentra Puregene Tissue Kit (QIAGEN, Venlo, Netherlands). Purified mtDNA was fragmented into approximately 100-bp fragments, and sequenced using HiSeq 2500 (Illumina, San Diego, CA), with chrM of the UCSC mm10 (http://hgdownload.cse.ucsc.edu/goldenPath/mm10/bigZips/chromFa.tar.gz) as a reference sequence. Mutations occurring at a frequency over 1% were analyzed to determine their effect on the encoded amino acid, and to check whether the mutation sites were homologous to sites of human pathogenic mtDNA mutation. Alignments of nucleic acid and amino acid sequences from mouse and human were performed for every gene and region using T-COFFEE (http://tcoffee.vital-it.ch/apps/tcoffee/do:regular).

### Native polyacrylamide gel electrophoreses (PAGE)

The crude mitochondrial fraction was isolated from cybrid homogenates by centrifugation (900 × *g* for 5 min). The supernatant was collected and re-centrifuged at 5,000 × *g* for 10 min. Isolated mitochondria (1 mg/mL) were solubilized at 4°C for 30 min with 1% digitonin in buffer (20 mM bis-Tris, 0.1 M NaCl, and 10% glycerol, pH 7.0), and centrifuged at 100,000 × *g* for 15 min. The resulting supernatants were subjected to electrophoresis. Electrophoresis was conducted as described before[17] using NativePAGE 3–12% Bis-Tris Protein Gels (Thermo Fisher Scientific, Waltham, MA). To detect respiratory complexes, proteins were transferred to a polyvinylidene fluoride (PVDF) membrane (Thermo Fisher Scientific). The membrane was blocked with 5% skim milk in TBS-T, and MitoProfile Total OXPHOS Blue Native WB Antibody Cocktail (at 1:250, MS603, Mitosciences, Eugene, OR) was used as the primary antibody, following the manufacturer’s recommendations. Horseradish peroxidase-linked anti-mouse IgG (at 1:3000, #7076, Cell Signaling Technology, Danvers, MA) was used as the secondary antibody, and incubated with ECL substrate (Thermo Fisher Scientific) in agreement with the manufacturer’s guidelines. Detection was performed using the ImageQuant LAS4000 (GE Healthcare, Little Chalfont, UK). To evaluate in-gel complex I activity, gels were incubated in assay buffer (2 mM Tris-HCl, 0.1 mg/mL NADH, and 2.5 mg/mL nitro blue tetrazolium chloride (NTB), pH 7.4).

### Quantification of proteins

Total protein was extracted from cybrids using the EzRIPA Lysis Kit (ATTO, Tokyo, Japan). SDS-PAGE was performed using NuPAGE 4–12% Bis-Tris gels (Thermo Fisher Scientific) and MOPS Running Buffer (Thermo Fisher Scientific). Then, the proteins were transferred to PVDF membrane. After blocking with a PVDF Blocking Reagent (TOYOBO, Osaka, Japan), membranes were incubated with the following primary antibodies: anti-NDUFA9 (ab14713, Abcam, Cambridge, UK), anti-MT-ND1 (ab181848, Abcam), anti-SDHA (#11998, Cell Signaling Technology), anti-COX IV (#4850, Cell Signaling Technology), anti-MT-COI (ab14705, Abcam), anti-Hsp60 (#12165, Cell Signaling Technology), anti-mtHsp70 (#3593, Cell Signaling Technology), anti-Tid1 (#4775, Cell Signaling Technology), anti-Lonp1 (NBP1-81734, Novus Biologicals, Littleton, CO), and anti-α–Tubulin (T-5168, Sigma-Aldrich). All primary antibodies described above were used at 1:1000 dilution. As the secondary antibodies, horse radish peroxidase-linked anti-mouse IgG (at 1:3000, #7076, Cell Signaling Technology) or anti-rabbit IgG (at 1:3000, #7074, Cell Signaling Technology) was used. The membranes were then incubated with ECL substrate (Thermo Fisher Scientific). Signal detection and quantification were performed using the ImageQuant LAS4000 (GE Healthcare) and MultiGauge software (FUJIFILM, Tokyo, Japan).

### Statistical analysis

The sample size of each test and statistical method used for data analysis are stated in each figure legend. Statistical analyses were conducted using GraphPad Prism 6.0 (GraphPad Software, La Jolla, CA). *P*-value of less than 0.05 was considered to indicate statistically significant differences between samples.

## Results

### Establishment of transmitochondrial cybrid cells harboring random mtDNA mutations

**Fig 1:**
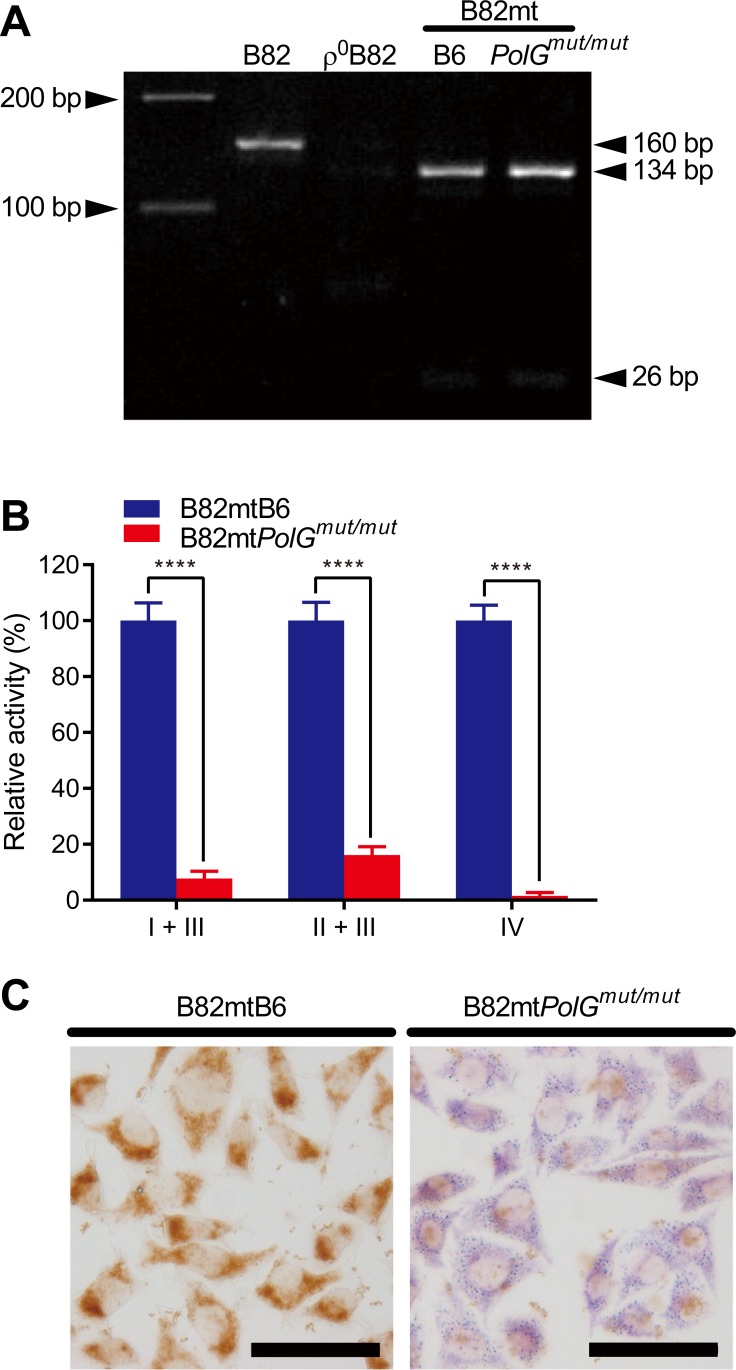
Establishment of transmitochondrial cybrid cells and their characterization. (**A**) Genotyping of mtDNA by PCR-restriction fragment length polymorphism confirmed the replacement of mtDNA in the constructed cybrids. Compared with the C57BL/6J mouse, mtDNA of the C3H/An mouse (the source of B82 cells) harbors G9348A polymorphism. Hence, the origin of mtDNA in the current study could be distinguished by detecting this strain-specific polymorphism (see methods for details). (**B**) Relative respiratory complex activities. Data are presented as the mean + S.D. ****P < 0.0001, according to the independent two-tailed *t*-test (*n* = 3). (**C**) COX/SDH staining of cybrid cells. Cells with normal COX activity stain brown, whereas cells with defective respiration stain blue. The images are representative of 3 replicates. Scale bar = 50 μm.

Previously, based on the original papers by Trifunovic *et al*. [7] and Kujoth *et al*. [8], we have introduced the D257A mutation into the *PolG* gene of C57BL/6J mouse and generated mtDNA mutator mouse [14]. As the mtDNA donor, we used a 10-month-old male *PolG*^mut/mut^ mouse, a generation 8 offspring of *PolG*^+/mut^-to-*PolG*^+/mut^ mating. Because successive generations of mice inherit maternal mtDNA mutations by germline transmission, it is thought that they harbor more mtDNA mutations than generation 1 offspring [18]. A 10-week-old C57BL/6J male mouse was used as a control. Platelets obtained from each mouse were fused with ρ^0^B82 cells [13], which are mtDNA-less cells from the B82 fibrosarcoma cell line derived from C3H/An mouse. Thus, B82mtB6 and B82mt*PolG*^mut/mut^ cybrids were established, accordingly (S1 Fig). Analysis of strain-specific mtDNA polymorphisms confirmed the replacement of C3H/An-type mtDNA by C57BL/6J-type mtDNA (Fig 1A). Measurements of respiratory complex activity revealed remarkable respiration defects in B82mt*PolG*^mut/mut^ cells (Fig 1B). Further, specific staining confirmed severe COX (complex IV) deficiency in B82mt*PolG*^mut/mut^ cells (Fig 1C). On the other hand, the staining indicated that the SDH (complex II) activity in B82mt*PolG*^mut/mut^ cells was rather up-regulated (Fig 1C). Thus, the reduced complex II + III activity shown in Fig 1B appeared to stem from a defect in complex III, and not a defect in complex II.

These observations indicated that the activities of respiratory complexes, except for complex II, in cells harboring mtDNA derived from the mutator mouse were severely impaired, and OXPHOS function was almost completely abolished in B82mt*PolG*^mut/mut^ cells.

### B82mt*PolG*^mut/mut^ cells harbor various mtDNA mutations at high frequency

**Fig 2:**
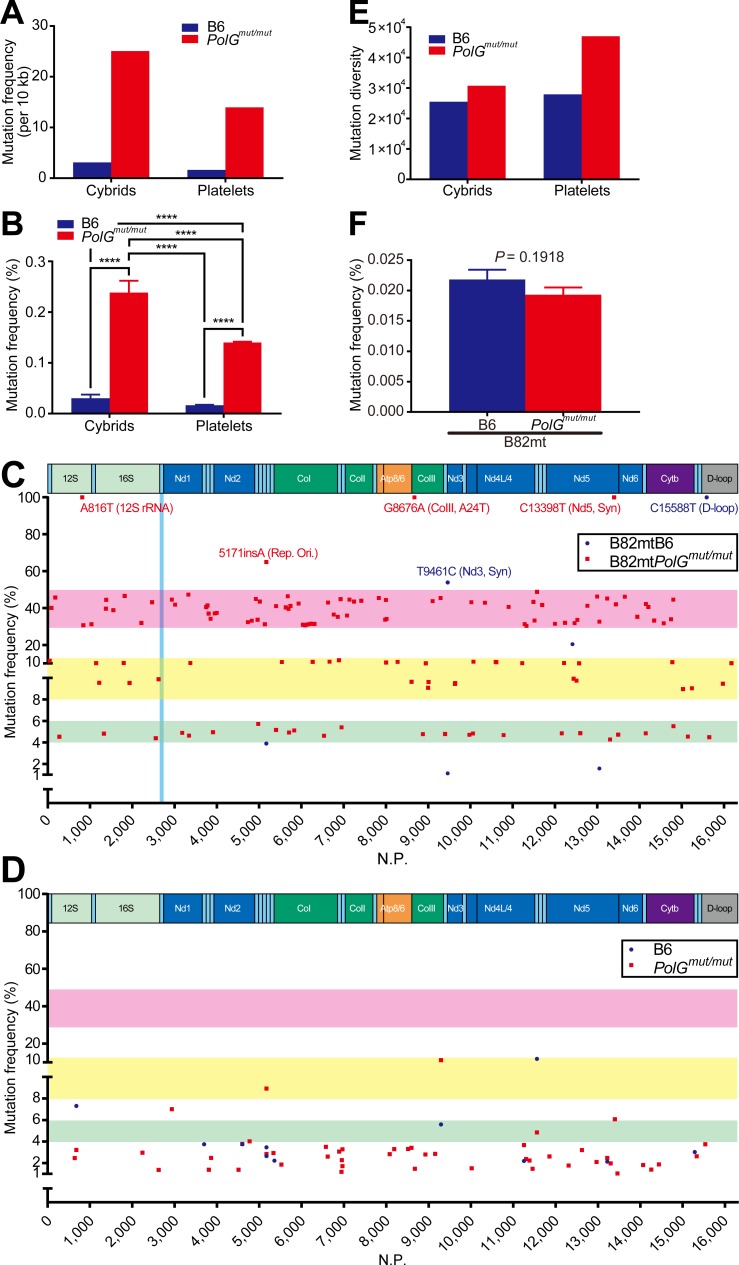
Deep-sequence analysis of the established cybrid cells. (**A**) Mutation frequency (per 10 kb) in each cybrid and platelet. The actual values are provided in Table 1. (**B**) Mutation frequency of each base in the entire mtDNA. Data are presented as the mean + S.E. ****P < 0.0001 by 1-way ANOVA. (*n* = 16,299). The actual values are provided in Table 1. Among all the detected mutations, only mutations occurring at a frequency of at least 1% in cybrid cells (**C**) and platelets (**D**) are shown. Green, yellow, and red bands indicate mutation ranges of 4–6%, 8–12%, and 30–50%, respectively. Blue band in (**C**) shows the *tRNA*^*Leu(UUR)*^ gene region. Rep. Ori., replication origin. (**E**) Comparison of diversity of mtDNA mutations. All types of substitution and insertion/deletion mutations were quantified independently, and total sums are shown (*e*.*g*., in the case of a base A mutated as A > G, A > T, and AinsAA, mutations for this base would be counted as 3). Note that platelets of *PolG*^mut/mut^ mice harbored more diverse mutations than B82mt*PolG*^mut/mut^ cells, despite a lower mutation frequency than that of B82mt*PolG*^mut/mut^ cells. (**F**) Mutation rates for each base in the *tRNA*^*Leu(UUR)*^ gene in cybrid cells. Data are presented as the mean + S.D. *P*-value calculated by using paired two-tailed *t*-test is indicated (*n* = 75).

We then extracted mtDNA molecules from each cybrid and platelets obtained from the littermate of each mtDNA-donor mouse of the cybrids, and analyzed by deep-sequencing. More than 2 million reads were analyzed. The average depth of coverage of the entire mtDNA sequence was over 12,000× (Table 1). Mutation analysis data are shown in S1 (cybrids) and S2 (platelets) Tables. The mutation frequency per 10 kb is shown in Table 1 and Fig 2A. As indicated in Table 1, Fig 2A and 2B, mtDNA molecules from B82mt*PolG*^mut/mut^ cells harbored approximately eight times more mutations than those of B82mtB6 control cells. The mutation frequency was also higher than that of platelets obtained from a littermate of an mtDNA-donor (Fig 2A and 2B). Mutations occurring at a frequency of at least 1% in cybrids and platelets are shown in Fig 2C and 2D, respectively. Only seven mutations fulfilling this criterion were identified in B82mtB6 cells, in comparison with 137 mutations in B82mt*PolG*^mut/mut^ cells. Regarding platelets, 11 and 48 mutations were found at more than 1% of frequency in B6 and *PolG*^mut/mut^ mice, respectively. This indicated that B82mt*PolG*^mut/mut^ cells harbored more mutations that occurred at high frequency than the control cells and platelets of mutator mice. Moreover, majority of the mutation rates of these 137 mutations were either approximately 5%, 10%, or 40% (Fig 2C). Notably, however, comparison of the diversity of mutations revealed that platelets from *PolG*^mut/mut^ mice harbored more diverse mutations than their cybrids, B82mt*PolG*^mut/mut^ cells (Fig 2E).

**Table 1:** Overview of sequence analysis.

		B82mt		Platelets	
		B6	*PolG*^mut/mut^	B6	*PolG*^mut/mut^
On-target read number		2,038,897	2,902,204	5,173,781	7,516,437
Depth average		12,442	17,701	31,565	45,824
Coverage rate	>3×	100.00	100.00	100.00	100.00
	>10×	100.00	100.00	100.00	100.00
	>20×	100.00	100.00	100.00	100.00
	>40×	99.99	100.00	100.00	100.00
Total analyzed bases		192,221,549	273,833,727	498,203,226	728,866,493
Total mutations*		59,960	686,532	81,320	1,018,148
Mutation frequency (bases/10 kb)		3.12	25.07	1.63	13.97
Mutation frequency for each base (%)		0.0307	0.2411	0.0165	0.1416

* The number of base substitutions and insertions/deletions.

Among mutations occurring at a frequency of >1% in cybrid cells, mutations mapped to protein-coding regions are listed in Table 2. Overall, 101 mutations were located in protein-coding regions; of these, 68 were non-synonymous mutations (67.3%). Mutations in the tRNA- or rRNA-coding, or non-coding regions are listed in Table 3. Among mutations listed in Tables 2 and 3, we attempted to identify mutations homologous to human pathogenic mtDNA mutations. Details of mutations occurring at homologous bases or affecting amino acids homologous to those of human pathogenic mutations listed in MITOMAP (https://mitomap.org), a human mitochondrial genome database, are shown in S3 Table.

**Table 2:** Mutations in protein-coding regions occurring at a frequency above 1% in cybrid cells.

Gene	N.P.	Ref.*	Mutation frequency		Most frequent change	Amino acid		Non-syn?**	Human N.P.
			B82mtB6	B82mt *PolG*^mut/mut^		Posi- tion	Change		
*Nd1*	2,934	C	0.044	44.522	>T	62	R > C	Yes	3,490
	3,010	T	0.014	41.861	>C	87	V > A	Yes	3,566
	3,181	T	0.015	4.904	>C	144	V > A	Yes	3,737
	3,328	C	0.054	47.286	>T	193	T > M	Yes	3,884
	3,340	C	0.055	4.646	>T	197	P > L	Yes	3,896
	3,373	T	0.015	10.176	>A	208	V > E	Yes	3,929
*Nd2*	3,953	T	0.029	37.104	>C	14	F > L	Yes	4,509
	3,997	A	0.037	37.327	DEL A	28	FrShift	Yes	4,553
	4,733	A	0.007	32.312	>T	274	N > Y	Yes	5,289
	4,826	T	0.030	33.102	>C	305	F > L	Yes	5,382
	4,922	C	0.059	44.905	>A	337	L > M	Yes	5,478
*CoI*	5,400	T	0.051	41.018	>C	25	W > R	Yes	5,976
	5,402	A	0.036	5.167	>G	25	W > W		5,978
	5,539	T	0.014	10.745	>C	71	M > T	Yes	6,115
	5,630	T	0.153	40.322	>C	101	S > S		6,206
	5,676	A	0.024	46.383	>C	117	M > L	Yes	6,252
	5,697	A	0.000	39.583	>G	124	T > A	Yes	6,273
	5,706	A	0.023	4.922	>G	127	T > A	Yes	6,282
	5,741	T	0.053	41.141	>C	138	H > H		6,317
	5,830	T	0.022	5.124	>C	168	I > T	Yes	6,406
	5,932	T	0.044	42.419	>C	202	L > P	Yes	6,508
	6,038	C	0.014	30.930	>T	237	F > F		6,614
	6,086	T	0.014	30.695	>C	253	I > I		6,662
	6,149	A	0.034	31.097	>G	274	V > V		6,725
	6,221	T	0.036	31.465	>C	298	D > D		6,797
	6,269	T	0.037	10.860	>C	314	I > I		6,845
	6,311	C	0.107	31.338	>T	328	H > H		6,887
	6,534	T	0.029	4.620	>C	403	F > S	Yes	7,110
	6,633	A	0.011	42.986	>G	436	M > V	Yes	7,209
	6,666	T	0.011	10.908	>C	447	Y > H	Yes	7,242
	6,765	C	0.026	36.510	>T	480	R > W	Yes	7,341
	6,861	A	0.014	35.214	>T	512	K > Stop	Yes	7,437
*CoII*	7,079	T	0.027	35.953	>C	23	F > L	Yes	7,652
	7,136	A	0.055	44.564	>G	42	I > V	Yes	7,709
	7,246	C	0.093	43.508	>G	78	L > L		7,819
	7,417	G	0.026	43.893	>A	135	L > L		7,990
*Atp8*	7,830	T	0.040	45.416	>C	22	I > T	Yes	8,430
*Atp6*	7,981	A	0.013	33.690	>G	19	I > V	Yes	8,581
	8,002	T	0.013	44.333	>C	26	F > L	Yes	8,602
	8,004	T	0.027	10.520	>C	26	F > F		8,604
	8,017	T	0.041	34.119	>C	31	F > L	Yes	8,617
	8,278	C	0.026	10.818	>T	118	R > W	Yes	8,878
*Atp6 /CoIII*	8,607	A	0.026	9.648	>G	227/1	Stop > Stop/M > M		9,207
*CoIII*	8,676	G	0.065	99.991	>A	24	A > T	Yes	9,276
	8,875	A	0.000	4.783	>G	90	E > G	Yes	9,475
	8,945	A	0.000	10.046	>G	113	G > G		9,545
	8,996	A	0.027	9.081	>C	130	P > P		9,596
	9,008	T	0.028	9.633	>G	134	T > T		9,608
	9,106	T	0.013	43.745	>C	167	I > T	Yes	9,706
	9,292	C	0.196	45.462	>T	229	S > L	Yes	9,892
*Nd3*	9,460	T	1.124	0.010	DEL T	1	FrShift	Yes	10,060
	9,461	T	53.846	0.015	>C	1	M > M		10,061
	9,632	A	0.024	9.456	>G	58	V > V		10,232
	9,638	T	0.000	9.529	>C	60	I > I		10,238
*Nd4L*	9,977	A	0.034	4.721	>G	34	E > G	Yes	10,570
	10,022	T	0.116	43.147	>A	49	L > Q	Yes	10,615
	10,060	A	0.000	4.839	>G	62	T > A	Yes	10,653
	10,072	T	0.016	10.906	>C	66	F > L	Yes	10,665
*Nd4*	10,342	A	0.014	42.844	>G	59	D > G	Yes	10,935
	10,596	A	0.000	10.864	>G	144	N > D	Yes	11,189
	10,610	T	0.000	10.638	>C	148	Y > Y		11,203
	10,786	T	0.021	4.693	>C	207	M > T	Yes	11,379
	10,903	T	0.020	40.613	>C	246	I > T	Yes	11,496
	11,227	T	0.030	10.145	>C	354	L > P	Yes	11,820
	11,283	A	0.035	31.306	>G	373	I > V	Yes	11,876
	11,323	T	0.020	30.334	>C	386	F > S	Yes	11,916
	11,489	A	0.014	43.339	>G	441	M > M		12,082
	11,518	C	0.043	33.252	>A	451	P > Q	Yes	12,111
*Nd5*	12,000	A	0.014	31.510	>G	87	I > V	Yes	12,595
	12,160	T	0.037	4.849	>C	140	L > P	Yes	12,755
	12,213	T	0.022	10.250	>C	158	W > R	Yes	12,808
	12,236	T	0.030	31.976	>C	165	N > N		12,831
	12,414	G	20.418	0.039	>A	225	A > T	Yes	13,009
	12,440	A	0.008	9.935	>G	233	L > L		13,035
	12,462	A	0.016	31.829	>G	241	T > A	Yes	13,057
	12,510	A	0.008	9.750	>G	257	I > V	Yes	13,105
	12,529	T	0.016	33.597	>C	263	F > S	Yes	13,124
	12,572	A	0.015	10.042	>G	277	M > M		13,167
	12,597	T	0.022	4.870	>C	286	L > L		13,192
	12,765	T	0.029	41.219	>A	342	C > S	Yes	13,360
	12,996	A	0.029	46.237	>G	419	T > A	Yes	13,591
	13,052	T	1.586	32.567	INS C	437	FrShift	Yes	13,647
	13,233	T	0.022	45.182	>C	498	F > L	Yes	13,828
	13,305	T	0.007	4.275	>C	522	F > L	Yes	13,900
	13,398	C	0.069	99.940	>T	553	L > L		13,993
	13,442	A	0.038	42.040	>G	567	S > S		14,037
	13,497	T	0.014	4.727	>C	586	L > L		14,092
*Nd6*	13,654	A	0.024	46.144	>T	139	I > M	Yes	14,254
	13,948	T	0.032	35.247	>C	41	L > L		14,551
*Cytb*	14,148	A	0.021	4.849	>G	2	T > A	Yes	14,750
	14,151	A	0.021	42.171	>G	3	N > D	Yes	14,753
	14,207	A	0.038	40.587	>G	21	L > L		14,809
	14,345	A	0.036	33.216	>T	67	T > T		14,947
	14,574	A	0.022	31.714	>G	144	T > A	Yes	15,176
	14,745	C	0.070	33.947	>T	201	H > Y	Yes	15,347
	14,777	A	0.036	10.641	>G	211	L > L		15,379
	14,797	T	0.022	44.566	>C	218	I > T	Yes	15,399
	14,802	T	0.007	5.513	>C	220	F > L	Yes	15,404
	15,030	T	0.022	8.968	>C	296	L > L		15,632
	15,145	T	0.008	4.545	>C	334	I > T	Yes	15,747
	15,241	T	0.014	9.047	>C	366	M > T	Yes	15,843

* Reference sequence.

** “Yes” if the mutation is a non-synonymous mutation.

**Table 3:** Mutations in rRNA and tRNA, or non-coding regions occurring at a frequency above 1% in cybrid cells.

Gene	N.P.	Ref*	Mutation frequency		Most frequent change	Human N.P.
			B82mt	B82mt		
			B6	*PolG*^*mut/mut*^		
*tRNA-Phe*	45	A	0.023	11.442	>G	623
*12S rRNA*	88	T	0.037	40.033	>C	667
	177	G	0.045	45.691	>A	754
	273	A	0.030	4.530	>C	852
	816	A	0.089	99.983	>T	1,392
	843	A	0.029	30.641	>G	1,420
*tRNA-Val*	1,033	A	0.015	31.182	>G	1,610
*16S rRNA*	1,144	T	0.007	10.113	>C	1,720
	1,218	A	0.007	9.562	>G	1,790
	1,328	A	0.015	4.820	>G	1,900
	1,373	A	0.053	39.642	>G	1,945
	1,381	A	0.044	44.471	>G	1,952
	1,551	A	0.007	38.831	>G	2,123
	1,795	A	0.028	10.260	>G	2,363
	1,819	C	0.074	46.556	>T	2,386
	1,935	T	0.023	9.536	>C	2,498
	2,215	G	0.023	31.925	>A	2,776
	2,468	T	0.069	43.184	>C	3,026
	2,555	T	0.022	4.398	>C	3,114
	2,620	T	0.015	9.891	>C	3,176
*tRNA-Ile*	3,747	T	0.014	40.502	>C	4,304
*tRNA-Gln*	3,777	A	0.029	41.356	>G	4,334
	3,807	T	0.023	37.029	>A	4,364
*tRNA-Met*	3,852	T	0.023	34.223	>C	4,409
	3,909	T	0.015	4.964	>A	4,465
*tRNA-Trp*	4,963	A	0.021	33.681	>G	5,525
	4,980	T	0.063	5.725	>C	5,543
*tRNA-Ala*	5,019	A	0.034	43.497	>G	5,588
*tRNA-Asn*	5,137	A	0.016	31.232	>T	5,705
Rep-Ori**	5,171	G	3.898	64.944	INS A	5,741
	5,172	A	3.161	0.867	DEL A	5,742
*tRNA-Ser(UCN)*	6,889	A	0.015	11.716	>G	7,465
	6,926	A	0.040	44.828	>G	7,502
*tRNA-Asp*	6,950	A	0.027	5.414	>G	7,526
*tRNA-Gly*	9,399	A	0.022	4.793	>G	9,999
*tRNA-His*	11,574	T	0.034	48.750	>C	12,167
*tRNA-Leu(CUN)*	11,696	T	0.014	41.697	>C	12,291
D-loop	15,588	C	99.931	0.020	>T	16,290
	15,651	T	0.030	4.484	>C	16,362
	15,974	A	0.024	9.471	>G	128
	16,176	T	0.022	10.159	>C	405

* Reference sequence.

** Replication origin.

Among mutations located in the protein-coding regions, no homologous mutations that constituted exact matches at base level to human pathogenic mutations were identified. However, three mutations resulted in changes of a homologous amino acid because they affected the same triplet codon as mutations in human. These mutations affected homologous amino acid but their effects (specific amino acid changes) were different from those observed in human disease cases (S3 Table). Therefore, although these three mutations were located at sites homologous to those of human pathogenic mutations, their effects on the function or structure of the encoded protein products were probably not homologous to human.

Among mutations in the tRNA-coding regions, three mutations occurred at bases homologous to those observed in human disease cases. Since both sites and base changes (the before and after status) corresponded to human cases, their effects on encoded products might be analogous to those in human diseases.

Next, we explored mutations homologous to confirmed pathogenic mutations in human mtDNA among mutations that occurred in the cybrids at a frequency below 1%. For all the confirmed pathogenic mutations listed in MITOMAP (last accessed: 17 December, 2018), whose pathogenicity has been strongly suggested, we surveyed the mutation rate of homologous mutations in the cybrids (S4 Table). As of December 2018, 83 confirmed pathogenic mutations were listed in MITOMAP. The frequency of the most frequently occurring homologous mutations was below 0.1%, indicating that these potential pathogenic mutations were contained at a very low frequency. Considering the *tRNA*^*Leu(UUR)*^ gene, known as a hotspot of human pathogenic mutations, the mutation rate of each base in B82mtB6 and B82mt*PolG*^mut/mut^ cells was similar (Fig 2F).

In summary, even though B82mt*PolG*^mut/mut^ cells contained various mutations at a higher frequency, not many mutations that occurred at a frequency of at least 1% were homologous to human pathogenic mutations, with respect to both site and quality.

### Abnormal respiratory complex formation in B82mt*PolG*^mut/mut^ cells

**Fig 3:**
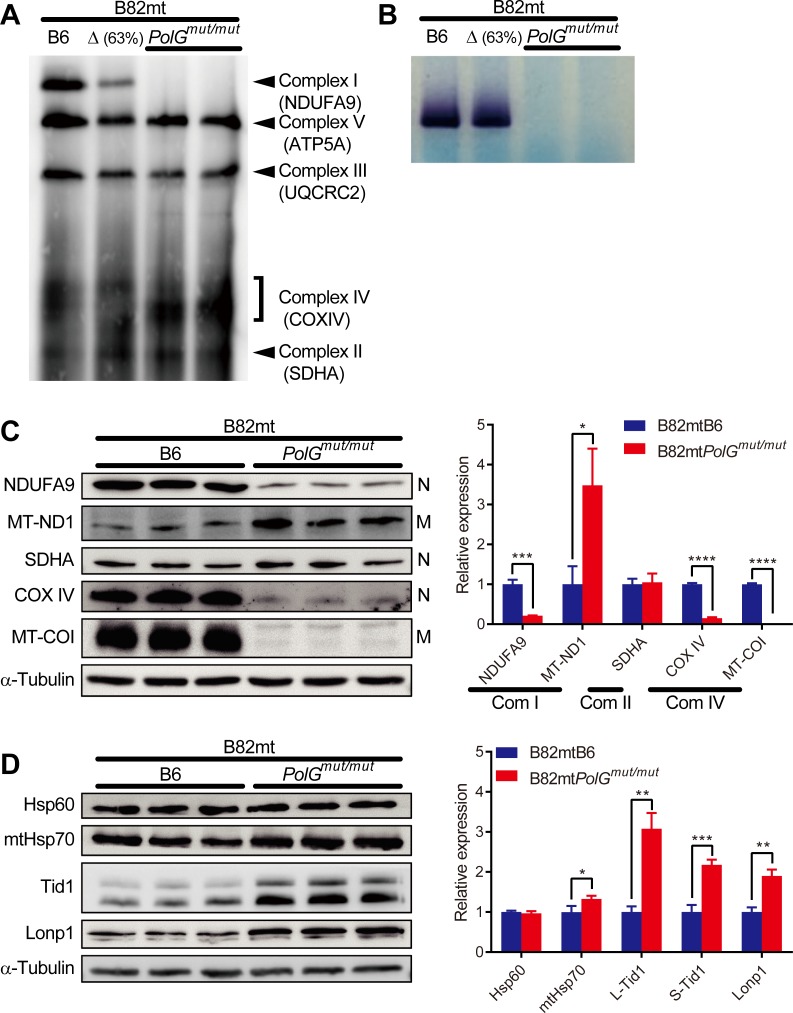
Abnormal respiratory complex formation in B82mt*PolG*^mut/mut^ cells. (**A**) Detection of the formed respiratory complexes by clear native PAGE. (**B**) In-gel evaluation of the activity of complex I. (**C**) Western blot analysis of the subunits of each respiratory complex (left) and band intensity quantification (right). Left: N and M refer to the nuclear DNA-encoded and mtDNA-encoded subunits, respectively. (**D**) Western blot analysis of mitochondrial chaperons, co-chaperons, and protease (left), and band intensity quantification (right). Data are presented as the mean + S.D. *P < 0.05, **P < 0.01, ***P < 0.001, ****P < 0.0001, according to an independent two-tailed *t*-test (*n* = 3).

Although B82mt*PolG*^mut/mut^ cells harbored few mutations homologous to human pathogenic mutations, they exhibited severe respiration defects. To determine the molecular mechanisms underpinning these respiration defects, we analyzed mitochondrial proteins by clear native PAGE [17] (Fig 3A). The experiment clearly demonstrated that complex I was dramatically reduced and almost undetectable in B82mt*PolG*^mut/mut^ cells. The reduction was more pronounced therein than in B82mtΔ cells, a cybrid with mtDNA with a large-scale deletion (ΔmtDNA) [3], which lacking genes encoding multiple subunits of complex I (Fig 3A). Considering the possibility of formation of a functional complex I in the absence of NDUFA9, the subunit used for the detection of complex I in clear native PAGE, we then evaluated the in-gel activity of complex I (Fig 3B). The activity of complex I was almost completely undetectable in this assay. On the other hand, complex II, all of which components are encoded by nuclear DNA, was not affected; the amounts of complex III and V were slightly reduced; and the mobility of complex IV exhibited a molecular mass shift to lower masses in B82mt*PolG*^mut/mut^ cells (Fig 3A).

We next evaluated the relative abundances of the subunits from complexes I and IV (the most affected complexes), and II (the unaffected complex). The data indicated that in addition to the changed abundances of mtDNA-encoded subunits, the abundances of nuclear DNA-encoded subunits of complexes I and IV were substantially altered (Fig 3C). By contrast, the abundances of complex II subunit were not significantly affected.

Since abnormal complex formation and unbalanced subunit levels were observed, we then examined the expression of representative chaperons and a protease, to determine whether the mitochondrial protein quality control system was experiencing stress. Although the expression of Hsp60 (also known as Hspd1), a major mitochondrial chaperon [19], was not significantly altered, the expression of mtHsp70 (also known as Grp75, Mortalin, or Hspa9), a key factor for protein folding in the matrix [20] and for protein import from the cytosol to the mitochondrion [21], was slightly up-regulated (Fig 3D). The expression of both, the long and short isoforms of Tid1 (also known as Dnaja3) [22], a co-chaperone for mtHsp70 and activator of its ATPase activity [23, 24], was dramatically increased. The expression of mitochondrial serine protease Lonp1 (also known as Prss15) was similarly elevated (Fig 3D).

## Discussion

In the current study, we investigated the possibility of using specific cybrids for mtDNA mutation accumulation, as a starting point for the investigation of links between specific mutations and disease. Deep-sequence analysis of the generated cybrid cells revealed that they harbored various mtDNA mutations at a higher frequency. Although the reported mutation rates in tissues of mutator mice depend on the methods or materials used (tissue type, animal age, and nuclear background used), they range, roughly, from several to dozen bases per 10 kb [7, 8, 18, 25]. In addition, we determined the mutation rate in platelets. The rate of 13.97 mutations per 10 kb (Table 1 and Fig 2A) was consistent with previous reports of other tissues. In contrast, the mutation rate in B82mt*PolG*^mut/mut^ cells was 25.07 bases per 10 kb over all mtDNA molecules (Table 1), which is several times higher than that reported in tissues and platelet [7, 8, 18, 25]. However, even more striking than the overall average mutation rate, several specific mutations accumulated at a high frequency in the generated cybrid cells. The frequency of occurrence of many mutations was over 40% in B82mt*PolG*^mut/mut^ cells (Fig 2C). These mutation frequencies were higher than any other rates for tissues of mutator mouse, such as the brain and heart [26], skeletal muscle [27], liver [28], and platelets reported here (Fig 2D). Mutations that occur at such high rates appear to be either fixed in mice over generations via the germline [18], or increase by random segregation [29] and/or clonal expansion [30, 31] after introduction into cultured cells.

Furthermore, most mutations occurring at a frequency above 1% in B82mt*PolG*^mut/mut^ cells converged around either approximately 5%, 10%, or 40% (Fig 2C). If each mutation rate increases or decreases randomly, such convergence would not occur. Thus, it appeared that the converged status was stable, and that some mechanisms might exists that maintain it. We suggest two hypothetical mechanisms. One involves the severity of pathogenesis of each mutation. Upper limits of mutation rates might exist in cells, depending on the mutation pathogenicity, split into roughly three stages. However, approximately 40% of mutations contained both synonymous and non-synonymous mutations, and mutations occurred at sites homologous to those of human pathogenic mutations were also contained. Approximately 5% of mutations also contained all of these mutations similarly. Thus, it cannot be simply assumed that pathogenicity of approximately 40% of mutations was low and that of approximately 5% of mutations was high. According to the other hypothetical mechanism, mutations that converged at similar mutation rates are linked on the same mtDNA molecule, with each mtDNA molecule containing approximately 5%, 10%, or 40% of the total mtDNA pool in B82mt*PolG*^mut/mut^ cells. In support of this hypothesis, it should be assumed that a heavily biased clonal expansion has occurred. In fact, it has been reported that in mutator mouse, tissue cells exhibiting respiration defects accumulate certain non-synonymous mutations at high frequency [16]. Further, clonal expansion was also observed after some drug treatments [32] or in tumor cells [33]. Taking into account these previous reports and the fact that the cybrids are tumor cells derived from fibrosarcoma, the possibility that clonal expansion plays a role in the convergence of mutation rates observed in B82mt*PolG*^mut/mut^ cells is conceivable. We thus developed a hypothesis related to the high mutation frequency following clonal expansion: Because platelets are very small cells, not all mutations we revealed in S2 Table would be contained in one or even a small number of platelets that fuse with ρ^0^B82 cells, indicating that a subpopulation of mutant mtDNAs would be introduced into ρ^0^B82 cells through cell fusion. Considering that B82 cells are considerably larger than platelets, the small number of mtDNAs introduced would replicate and increase in number. It can be considered that this process is similar to that of mtDNA bottlenecking during oocyte development. Previously, it was proposed that biased replication of a subpopulation of mtDNAs results in the genetic bottleneck of mtDNA [34]. This biased replication could occur in the generated cybrid cells and induces the clonal expansion of some specific mtDNA molecules without any selection, as ρ^0^B82 cells can harbor severe pathogenicities induced by predominant accumulation of pathogenic mtDNAs, owing to their respiration defect resistance (S2 Fig). In animal tissues or organs, no mtDNA-less cells are present except for terminally differentiated erythrocytes, and mitochondrial respiration-null cells would not survive. In contrast, ρ^0^B82 cells can survive without mtDNA and adapt to and/or resist a mitochondrial respiration-free environment. We consider that this strong resistance to respiration defects and biased replication of mtDNA enable pathogenic mtDNA mutations to accumulate at a high frequency. The higher frequency but lower diversity of mtDNA mutations in our cybrid cells compared with parental platelets (Fig 2A–2E) also supports our hypothesis related to biased replication of a subpopulation of mtDNA in parental platelets (S2 Fig).

At the same time, notably, whereas B82mt*PolG*^mut/mut^ cells harbored various mutations, including non-synonymous mutations, at a high frequency, almost no mutations homologous to human pathogenic mutations were observed (S3 Table). When the entire mtDNA molecules were compared, it was apparent that the mtDNA mutation rate in B82mt*PolG*^mut/mut^ cells was significantly higher than that in B82mtB6 cells (Table 1 and Fig 2A and 2B). However, when the comparison region was restricted to the *tRNA*^*Leu(UUR)*^ gene, the hotspot of human pathogenic mtDNA mutations, the mutation rates for each base in B82mtB6 and B82mt*PolG*^mut/mut^ cells were not significantly different (Fig 2E), indicating that the frequency of mutations in this region was not increased by *PolG* mutation. Focusing on a more specific site, the A2689G mutation, a mutation homologous to human A3243G mutation, which is the most frequent mutation observed in human mitochondrial diseases, no such reads were detected among 13,997 reads in B82mtB6. Even in the B82mt*PolG*^mut/mut^ cells, only two such reads were identified among 19,201 reads (S1 Table). These observations suggested that mutation hotspot regions in human mtDNA and hotspot regions induced by *PolG*^mut/mut^ in mouse mtDNA are dissimilar.

Although we did not identify mutations homologous to confirmed human pathogenic mutations that occurred at a high frequency in B82mt*PolG*^mut/mut^ cells, these cells exhibited severe respiration defects (Fig 1B and 1C). Abnormal respiratory complex formation appeared to be involved in these respiration defects. In the most affected complex I, whereas drastic changes in the expression of complex subunits were observed, not all of them were completely lost (Fig 3C). However, the formation and function of complex I were almost entirely abolished (Fig 3A and 3B). Although abnormal respiratory complex formation has been reported in mutator mouse tissues [35], almost complete disappearance of complex I observed in the cybrid cells in the current study was much more severe than that observed in mouse tissues. These exceedingly severe abnormalities were not lethal to the cultured cell line, in contrast with mouse tissues or organs. Furthermore, the increased expression of chaperons, co-chaperons, and a protease in the B82mt*PolG*^mut/mut^ cells (Fig 3D) probably reflected accelerated protein degradation by proteases because of the shortened lifespan of respiratory complexes incorporating abnormal subunits, and activated protein transport from the cytosol to the mitochondrial matrix to recruit subunits to replace the ones that had been degraded.

Analyses presented in the current study revealed that a cultured cell line can indeed harbor various mtDNA mutations that occur at a high frequency, and can be maintained regardless of the induced severe respiration defects, which are not viable in mouse tissues. On the other hand, despite the fact that the generated cybrids harbored various mutations, few mutations homologous to human pathogenic mutations were identified. Therefore, it might be challenging to use the generated cybrids as a source of mutations for the development of novel mouse models of specific human diseases. However, cultured cells that harbor frequently occurring harmful mutations are very useful tools for concentrating mtDNA mutations. Some of the mutations identified in the current study might be pathogenic mutations that are common predisposing factors for human diseases, even if the mutation sites are not entirely consistent with human. Importantly, since ρ^0^B82 cells, the mtDNA recipient cells used in this study, express wildtype *PolG*, accumulation of *de novo* mtDNA mutations rarely occurred in re-populated mtDNA molecules after fusion. Our sequence analysis suggested that at least a portion of re-populated mtDNAs were clonally expanded from the small population of introduced mtDNAs (S2 Fig). Many mutations were linked on the same mtDNA molecule, and as a result, the effects of individual mutations in this condition in our current cybrid cells cannot be evaluated; however, controlling the mutation load in parental platelets by generation, age, or mtDNA refresh through mating of male mutator mice with female wild-type mice [18], may enable control of mtDNA populations in established cybrid cells. When the small population of introduced mtDNAs harbor useful mutations, the mutations can be concentrated in generated cybrid at levels that would be fatal in mouse tissue. Ultimately, these mutations can be introduced into mouse at appropriate mutation rate [36], and novel mouse models of mtDNA-based diseases can be generated. The generated mouse models may facilitate understanding of the mechanism of diverse disease phenotypes due to mtDNA mutations.

## Supporting information

S1 Fig Scheme for the establishment of transmitochondrial cybrids. For the experiment, ρ^0^B82 cells and platelets obtained from mice were used as mtDNA recipients and donors, respectively. Recipient and donor cells were fused using the polyethylene glycol method, and the resultant transmitochondrial cybrids were selected by culturing in a selective medium (see Materials and Methods for details).
(EPS)

S2 Fig Schematic illustration of the predicted process by which mtDNA mutations are concentrated at high frequency. The platelets contain diverse mtDNA mutations as a population, but “real parental” platelets, which fuse with ρ^0^B82 cells, harbor only a portion thereof. Because ρ^0^B82 cells are larger than platelets, the introduced mtDNA molecules replicate and increase in number after cell fusion. Because of the biased replication of mtDNA, a subpopulation of mtDNA molecules dominantly increases, and mutations on those molecules also increase simultaneously. Although accumulation of various mtDNA mutations at high frequency induces severe respiration defects, the generated cybrid cells can survive and proliferate because of their resistance to the mitochondrial respiration-null condition, which was acquired during the ρ^0^ state. As a result, a subpopulation of mutations in the parental platelets is concentrated, resulting in a higher mutation frequency, but the diversity of mutations is lower than that of parental platelets.
(EPS)

S1 Table Mutation analysis of cybrid cells. (XLSX)

S2 Table Mutation analysis of platelets. (XLSX)

S3 Table Mutations homologous to human pathogenic mutations occurring at a frequency above 1% in B82mt*PolG*^*mut/mut*^ cybrid cells. (XLSX)

S4 Table Mutations homologous to human pathogenic mutations occurring at a frequency under 1% in B82mt*PolG*^*mut/mut*^ cybrid cells. (XLSX)

## References

[1] Wallace DC. Mitochondrial diseases in man and mouse. Science. 1999;283(5407):1482–8. 10066162 10.1126/science.283.5407.1482

[2] Tuppen HA, Blakely EL, Turnbull DM, Taylor RW. Mitochondrial DNA mutations and human disease. Biochim Biophys Acta. 2010;1797(2):113–28. 10.1016/j.bbabio.2009.09.005 19761752

[3] Inoue K, Nakada K, Ogura A, Isobe K, Goto Y, Nonaka I, et al Generation of mice with mitochondrial dysfunction by introducing mouse mtDNA carrying a deletion into zygotes. Nat Genet. 2000;26(2):176–81. 10.1038/82826 11017072

[4] Kasahara A, Ishikawa K, Yamaoka M, Ito M, Watanabe N, Akimoto M, et al Generation of trans-mitochondrial mice carrying homoplasmic mtDNAs with a missense mutation in a structural gene using ES cells. Hum Mol Genet. 2006;15(6):871–81. 10.1093/hmg/ddl005 16449238

[5] Yokota M, Shitara H, Hashizume O, Ishikawa K, Nakada K, Ishii R, et al Generation of trans-mitochondrial mito-mice by the introduction of a pathogenic G13997A mtDNA from highly metastatic lung carcinoma cells. FEBS Lett. 2010;584(18):3943–8. 10.1016/j.febslet.2010.07.048 20674568

[6] Shimizu A, Mito T, Hayashi C, Ogasawara E, Koba R, Negishi I, et al Transmitochondrial mice as models for primary prevention of diseases caused by mutation in the tRNA(Lys) gene. Proc Natl Acad Sci U S A. 2014;111(8):3104–9. 10.1073/pnas.1318109111 24510903 PMC3939884

[7] Trifunovic A, Wredenberg A, Falkenberg M, Spelbrink JN, Rovio AT, Bruder CE, et al Premature ageing in mice expressing defective mitochondrial DNA polymerase. Nature. 2004;429(6990):417–23. 10.1038/nature02517 15164064

[8] Kujoth GC, Hiona A, Pugh TD, Someya S, Panzer K, Wohlgemuth SE, et al Mitochondrial DNA mutations, oxidative stress, and apoptosis in mammalian aging. Science. 2005;309(5733):481–4. 10.1126/science.1112125 16020738

[9] Stewart JB, Freyer C, Elson JL, Wredenberg A, Cansu Z, Trifunovic A, et al Strong purifying selection in transmission of mammalian mitochondrial DNA. PLoS Biol. 2008;6(1):e10 10.1371/journal.pbio.0060010 18232733 PMC2214808

[10] Goto Y, Nonaka I, Horai S. A mutation in the tRNA(Leu)(UUR) gene associated with the MELAS subgroup of mitochondrial encephalomyopathies. Nature. 1990;348(6302):651–3. 10.1038/348651a0 2102678

[11] Ono T, Isobe K, Nakada K, Hayashi JI. Human cells are protected from mitochondrial dysfunction by complementation of DNA products in fused mitochondria. Nat Genet. 2001;28(3):272–5. 10.1038/90116 11431699

[12] King MP, Attardi G. Human cells lacking mtDNA: repopulation with exogenous mitochondria by complementation. Science. 1989;246(4929):500–3. 2814477 10.1126/science.2814477

[13] Inoue K, Takai D, Hosaka H, Ito S, Shitara H, Isobe K, et al Isolation and characterization of mitochondrial DNA-less lines from various mammalian cell lines by application of an anticancer drug, ditercalinium. Biochem Biophys Res Commun. 1997;239(1):257–60. 9345305 10.1006/bbrc.1997.7446

[14] Mito T, Kikkawa Y, Shimizu A, Hashizume O, Katada S, Imanishi H, et al Mitochondrial DNA mutations in mutator mice confer respiration defects and B-cell lymphoma development. PLoS One. 2013;8(2):e55789 10.1371/journal.pone.0055789 23418460 PMC3572082

[15] Ishikawa K, Hayashi J. Generation of mtDNA-exchanged cybrids for determination of the effects of mtDNA mutations on tumor phenotypes. Methods Enzymol. 2009;457:335–46. 10.1016/S0076-6879(09)05019-8 19426877

[16] Vermulst M, Wanagat J, Kujoth GC, Bielas JH, Rabinovitch PS, Prolla TA, et al DNA deletions and clonal mutations drive premature aging in mitochondrial mutator mice. Nat Genet. 2008;40(4):392–4. 10.1038/ng.95 18311139

[17] Wittig I, Carrozzo R, Santorelli FM, Schagger H. Functional assays in high-resolution clear native gels to quantify mitochondrial complexes in human biopsies and cell lines. Electrophoresis. 2007;28(21):3811–20. 10.1002/elps.200700367 17960833

[18] Ross JM, Stewart JB, Hagstrom E, Brene S, Mourier A, Coppotelli G, et al Germline mitochondrial DNA mutations aggravate ageing and can impair brain development. Nature. 2013;501(7467):412–5. 10.1038/nature12474 23965628 PMC3820420

[19] Ostermann J, Horwich AL, Neupert W, Hartl FU. Protein folding in mitochondria requires complex formation with hsp60 and ATP hydrolysis. Nature. 1989;341(6238):125–30. 10.1038/341125a0 2528694

[20] Kaul SC, Deocaris CC, Wadhwa R. Three faces of mortalin: a housekeeper, guardian and killer. Exp Gerontol. 2007;42(4):263–74. 10.1016/j.exger.2006.10.020 17188442

[21] Schneider HC, Berthold J, Bauer MF, Dietmeier K, Guiard B, Brunner M, et al Mitochondrial Hsp70/MIM44 complex facilitates protein import. Nature. 1994;371(6500):768–74. 10.1038/371768a0 7935837

[22] Syken J, De-Medina T, Munger K. TID1, a human homolog of the Drosophila tumor suppressor l(2)tid, encodes two mitochondrial modulators of apoptosis with opposing functions. Proc Natl Acad Sci U S A. 1999;96(15):8499–504. 10411904 10.1073/pnas.96.15.8499 PMC17545

[23] Bukau B, Horwich AL. The Hsp70 and Hsp60 chaperone machines. Cell. 1998;92(3):351–66. 9476895 10.1016/s0092-8674(00)80928-9

[24] Cyr DM, Langer T, Douglas MG. DnaJ-like proteins: molecular chaperones and specific regulators of Hsp70. Trends Biochem Sci. 1994;19(4):176–81. 8016869 10.1016/0968-0004(94)90281-x

[25] Khrapko K, Kraytsberg Y, de Grey AD, Vijg J, Schon EA. Does premature aging of the mtDNA mutator mouse prove that mtDNA mutations are involved in natural aging? Aging cell. 2006;5(3):279–82. 10.1111/j.1474-9726.2006.00209.x 16842501

[26] Williams SL, Huang J, Edwards YJ, Ulloa RH, Dillon LM, Prolla TA, et al The mtDNA mutation spectrum of the progeroid Polg mutator mouse includes abundant control region multimers. Cell Metab. 2010;12(6):675–82. 10.1016/j.cmet.2010.11.012 21109200 PMC3175596

[27] Dillon LM, Williams SL, Hida A, Peacock JD, Prolla TA, Lincoln J, et al Increased mitochondrial biogenesis in muscle improves aging phenotypes in the mtDNA mutator mouse. Hum Mol Genet. 2012;21(10):2288–97. 10.1093/hmg/dds049 22357654 PMC3335313

[28] Ni T, Wei G, Shen T, Han M, Lian Y, Fu H, et al MitoRCA-seq reveals unbalanced cytocine to thymine transition in Polg mutant mice. Sci Rep. 2015;5:12049 10.1038/srep12049 26212336 PMC4648470

[29] Jenuth JP, Peterson AC, Fu K, Shoubridge EA. Random genetic drift in the female germline explains the rapid segregation of mammalian mitochondrial DNA. Nat Genet. 1996;14(2):146–51. 10.1038/ng1096-146 8841183

[30] Yao YG, Ogasawara Y, Kajigaya S, Molldrem JJ, Falcao RP, Pintao MC, et al Mitochondrial DNA sequence variation in single cells from leukemia patients. Blood. 2007;109(2):756–62. 10.1182/blood-2006-01-011007 16946307 PMC1785100

[31] Elson JL, Samuels DC, Turnbull DM, Chinnery PF. Random intracellular drift explains the clonal expansion of mitochondrial DNA mutations with age. Am J Hum Genet. 2001;68(3):802–6. 10.1086/318801 11179029 PMC1274494

[32] Payne BA, Wilson IJ, Hateley CA, Horvath R, Santibanez-Koref M, Samuels DC, et al Mitochondrial aging is accelerated by anti-retroviral therapy through the clonal expansion of mtDNA mutations. Nat Genet. 2011;43(8):806–10. 10.1038/ng.863 21706004 PMC3223397

[33] Gekeler J, Zsurka G, Kunz WS, Preuss SF, Klussmann JP, Guntinas-Lichius O, et al Clonal expansion of different mtDNA variants without selective advantage in solid tumors. Mutat Res. 2009;662(1–2):28–32. 10.1016/j.mrfmmm.2008.11.020 19114048

[34] Wai T, Teoli D, Shoubridge EA. The mitochondrial DNA genetic bottleneck results from replication of a subpopulation of genomes. Nat Genet. 2008;40(12):1484–8. 10.1038/ng.258 19029901

[35] Edgar D, Shabalina I, Camara Y, Wredenberg A, Calvaruso MA, Nijtmans L, et al Random point mutations with major effects on protein-coding genes are the driving force behind premature aging in mtDNA mutator mice. Cell Metab. 2009;10(2):131–8. 10.1016/j.cmet.2009.06.010 19656491

[36] Ishikawa K, Kasahara A, Watanabe N, Nakada K, Sato A, Suda Y, et al Application of ES cells for generation of respiration-deficient mice carrying mtDNA with a large-scale deletion. Biochem Biophys Res Commun. 2005;333(2):590–5. 10.1016/j.bbrc.2005.05.155 15953585

